# High-throughput targeted screening in triple-negative breast cancer cells identifies Wnt-inhibiting activities in Pacific brittle stars

**DOI:** 10.1038/s41598-017-12232-7

**Published:** 2017-09-20

**Authors:** Artem Blagodatski, Vsevolod Cherepanov, Alexey Koval, Vladimir I. Kharlamenko, Yuri S. Khotimchenko, Vladimir L. Katanaev

**Affiliations:** 1 School of Biomedicine, Far Eastern Federal University, Vladivostok, Russian Federation; 2 National Scientific Center for Marine Biology, Far Eastern Branch of Russian Academy of Sciences, Vladivostok, Russia; 3 Department of Pharmacology and Toxicology, Faculty of Biology and Medicine, University of Lausanne, Lausanne, 1011 Switzerland

## Abstract

Pro-proliferative oncogenic signaling is one of the hallmarks of cancer. Specific targeting of such signaling pathways is one of the main approaches to modern anti-cancer drug discovery, as opposed to more traditional search for general cytotoxic agents. Natural products, especially from marine sources, represent a largely untapped source of chemical diversity, which so far have mostly been screened for cytotoxicity. Here we present a pioneering pipeline of high-throughput screening of marine-based activities targeted against the Wnt signaling pathway, which is one of the key factors in oncogenic transformation, growth and metastasis in different cancers, including the devastating triple-negative breast cancer (TNBC) currently lacking any targeted therapies. This pipeline consisted of collection and characterization of numerous invertebrates during the SokhoBio expedition to the Kuril Basin in North Pacific, preparation of extracts from these specimen, and their screening in dedicated assays monitoring Wnt signaling in TNBC cells. This approach yielded a number of promising hits, including highly specific anti-Wnt activities targeting multiple levels within the Wnt pathway from *Ophiura irrorata* and other Pacific brittle stars.

## Introduction

Of all marketed drugs, about 40% originate from natural compounds (either directly or through synthetic modifications), and this share grows to ca. 50% when looking at small molecule drugs^1^. Of those, marine-derived drugs represent a minority, as their exploitation started only in the 1970s, but are of a high promise, as the chemical novelty and diversity of marine-derived natural products exceeds that of terrestrial sources^2,3^. Continuing exploration of the biodiversity of marine environments will inevitably bring more drugs originating from marine compounds.

Among different disease conditions, cancer has been the target of extensive screenings of natural products as the potential source of anti-cancer drugs. These efforts have resulted in appearance of several anticancer chemotherapeutic agents, such as camptothecines, anthracyclines, taxanes and vinca alcaloids, either originating directly from plants or bacteria or being synthetic derivatives of natural compounds^4^. The search for marine-derived drugs, although started later, has already resulted in some promising anti-cancer agents, such as Eribulin (Halaven), Trabectedin (Yondelis) and Brentuximab vedotin (Adcetris), marketed against different forms of cancer, such as breast cancer and liposarcoma, soft tissue sarcoma, and lymphomas^5^.

However, a common feature of most natural product-originating chemotherapeutics is their general cytotoxicity: they more strongly affect metabolically active and proliferating cells such as most tumor cells, but are also toxic for the healthy cells of the patient. In contrast, development of targeted therapies is the new focus of anti-cancer research of the past decades. Such targeted drugs are selective for the cancer cells and have less or (ideally) zero toxicity against healthy tissues. Among the hallmarks of cancer, excessively activated signaling pathways that are responsible for continuing proliferation of the cancer cells^6^ have been one of the major targets for the anti-cancer drug discovery, giving rise to several targeted drugs, such as vismodegib – a selective inhibitor of the Smoothened receptor suppressing aberrant Hedgehog signaling in the treatment of basal cell carcinoma^7^.

Several oncogenic signaling pathways exist. In breast cancer (BC) as an example, three major subtypes of the disease are identified based on the type of the over-activated signaling pathway driving excessive cell proliferation^8^. The first BC subtype is estrogen-receptor positive, receives the command for excessive proliferation from over-activation of the estrogen signaling, and is susceptible to targeted anti-hormonal therapies such as tamoxifen^9^. The second subtype relies on over-production of the receptor tyrosine kinase (RTK) HER2 and resulting over-activation of the RTK pathway for excessive cell proliferation; the targeted treatment with anti-HER2 monoclonal antibodies, marketed under the trastuzumab (Herceptin) trade names, is typically applied against this BC subtype^10^.

The third BC subtype is negative in expression of estrogen and HER2 receptors, as well as progesterone receptor, as is therefore called triple-negative breast cancer (TNBC). Being insensitive to the existing targeted therapies, TNBC accounts for more than half of all BC-related deaths, taking away some 200000 lives annually on the global scale^11,12^. It is widely accepted that one of the most common sources of the pro-proliferation signal in TNBC cells is the over-activation of the Wnt signaling pathway^13–15^. The canonical Wnt signaling pathway plays critical roles in embryonic development^16^ but is also important for the adult life of all metazoans including humans. The pathway controls cell proliferation and differentiation, and regulates proliferation and self-renewal of various stem cells as well as regenerative processes^17^. Aberrant activation or hyper-activation of the Wnt pathway can lead to carcinogenesis in several adult organs, most notably colon^18^ and breast^19^. Regarding the TNBC, the Wnt/β-catenin signaling, along with other developmental signaling pathways including Cripto-1 and Notch/CSL, is of special importance for the regulation of cancer stem cells and for the therapy resistance^15^. Dysregulation of Wnt signaling in TNBC is not only one of the driving forces of initial transformation^20^ but also promotes cell migration, colony formation, stem-like features and chemoresistance, playing important roles also in the later stages of tumor progression^14^. Detailed studies on gene expression profiles have subdivided the TNBC into six subtypes, all of which demonstrate aberrant overexpression of certain Wnt-associated genes^21^. Taken together, the modern knowledge suggests that in development of targeted treatments against TNBC, Wnt pathway inhibitors should play an essential though not an exclusive role^14^. The Wnt pathway is a complex signaling cascade. In humans, it operates with 19 types of Wnt proteins as ligands and 10 types of Frizzled proteins as receptors. The downstream components of the pathway involve a co-receptor LRP 5/6, G-proteins, a multiprotein β-catenin destruction complex (containing Axin, APC, glycogen synthase kinase 3β (GSK3β) and casein kinase), β-catenin itself (which serves as a cofactor of the key transcription factor TCF inducing expression of the Wnt target genes) and many more cofactors of the latter. Taken together, it gives a variety of targets for the inhibition of the pathway^22,23^.

In TNBC, overexpression of Wnt ligands, the receptor Frizzled7, and the co-receptor LRP6 has been described^24–26^. After that, development of agents targeting the Wnt signaling pathway as future anti-TNBC drugs (as well as drugs against other Wnt-dependent cancers) has been initiated. One approach involves development of human antibodies against upstream extracellular components of the pathway, such as an antibody against the cysteine-rich (extracellular) domain of Frizzled7, which later showed cross-reactivity with 4 other Frizzleds but efficiency against several solid cancers *in vitro* ^27^; phase I clinical studies are currently in place. Screening of libraries of synthetic small molecules is another approach to derive Wnt signaling inhibitors^28^. Several hit compounds have been identified, among them the molecule XAV939 inhibiting tankyrase – a multifunctional enzyme among other things regulating the activity of the β-catenin-destruction complex^29^. Rational drug design and *in silico* screening is also applicable to the Wnt pathway, especially after the recent description of the crystal structure of a Wnt-Frizzled complex^30^; an example of such *in silico* analysis is our recent identification of an anti-leprosy drug clofazimine as a potent inhibitor of the Wnt pathway and growth of TNBC cells *in* *vitro* ^31^.

Despite these efforts, no anti-Wnt signaling agents have so far made it to the clinic nor even advanced clinical trials^14^. As the demand for such drugs to treat TNBC and other Wnt-dependent cancers is urgent, other approaches to develop Wnt signaling inhibitors should also be considered. We here describe a paradigm of screening for anti-Wnt, anti-TNBC activities from marine sources. Collection of marine invertebrate species performed during the SokhoBio expedition^32^ and extract preparation is hereby followed by high-throughput screening for inhibitors of the Wnt pathway in TNBC cells. This approach has resulted in identification of promising activities from brittle stars of North Pacific and broad characterization of their mode of action within the Wnt pathway. Other interesting Wnt inhibitory and activatory activities are also identified in other species. Upscaling and further development of this paradigm may promote the discovery of lead compounds to develop treatments against the Wnt-dependent cancers.

## Results

**Figure 1:**
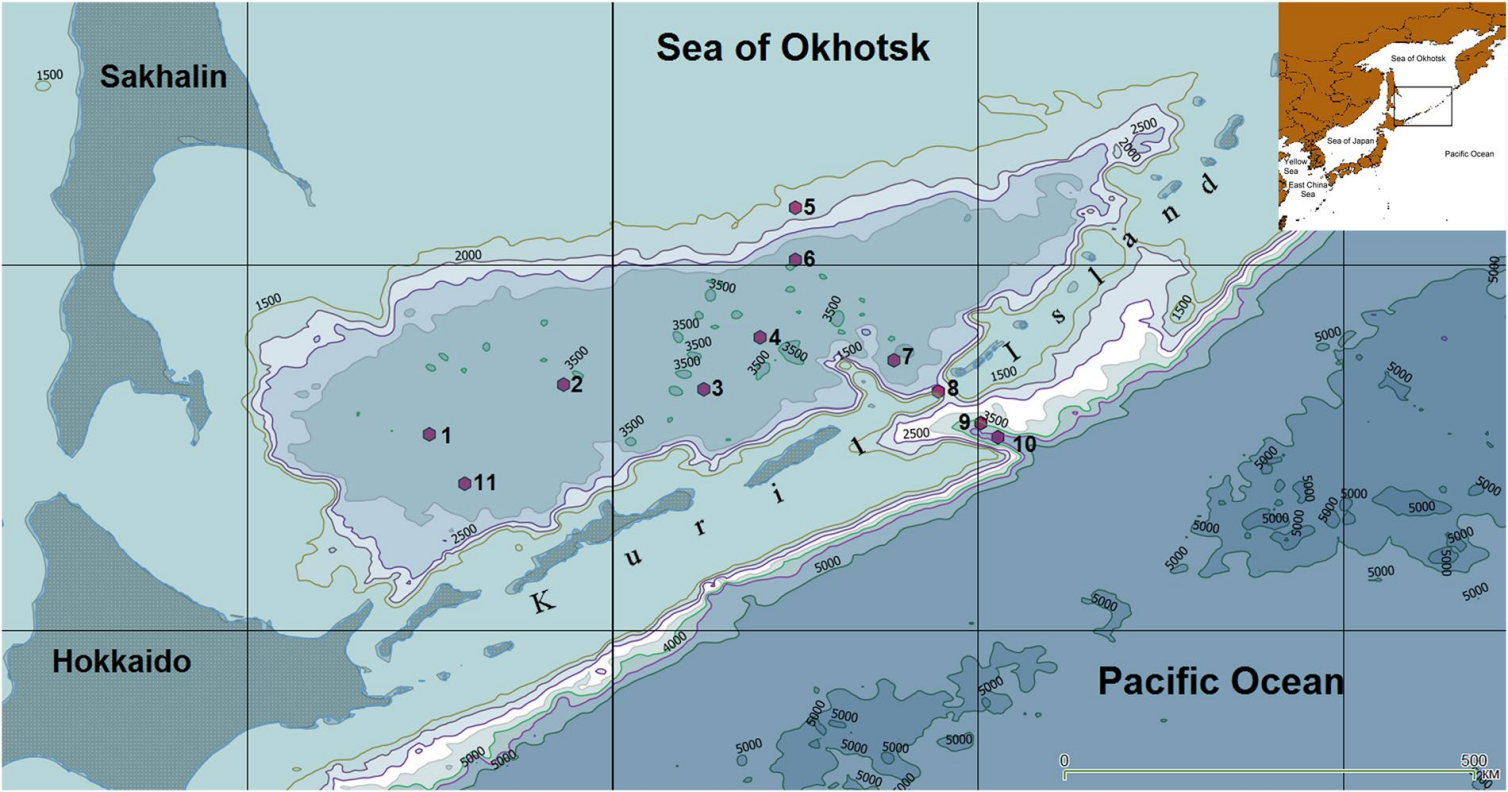
Map of the SokhoBio expedition of the research vessel “Akademik M.A. Lavrentiev”, departing from and returning to Vladivostok, from 5^th^ of June to 6^th^ of August 2015 in the Kuril Basin of the Sea of Okhotsk, northwestern Pacific Ocean. 1 to 11 illustrates the eleven stations where specimens were collected (see Supplementary Table S1). Sea depths are indicated. Insert in the upper right corner places the expedition region into a bigger map of the North Pacific area. The map was drawn using the QGIS 2.16.1-Nødebo - Quantum GIS Geographic Information System, Open Source Geospatial Foundation Project by the “Quantum GIS Development Team” (2016) - a free open source geoinformation system software available under following web address: http://www.qgis.org/eng/site.

**Figure 2:**
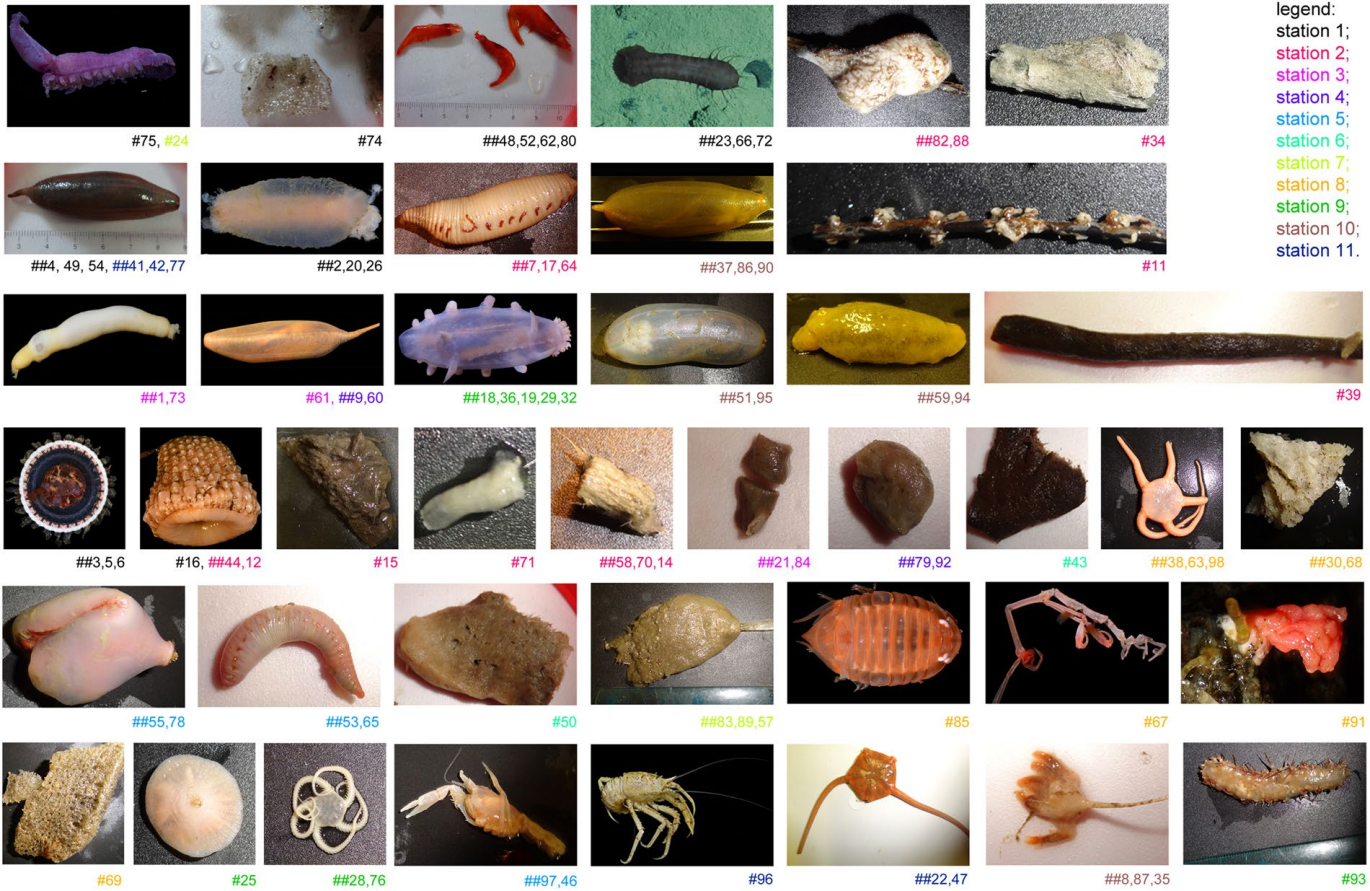
Photographs (not in scale) of representatives of the ca. 80 invertebrate specimens analyzed in this study. Specimen number(s) is provided below each image. Color coding refers to each of the eleven stations, where the specimens were collected (see Supplementary Table S1 and Fig. 1).

During the SokhoBio expedition^32^ of the research vessel “Akademik M.A. Lavrentiev” from 5^th^ of June to 6^th^ of August 2015, invertebrate organisms were collected in the Kuril Basin of the Sea of Okhotsk, northwestern Pacific Ocean, at the depths ranging from 1700 to 4750 m (Fig. 1 and Supplementary Table S1). Specimen from the following invertebrate groups were collected (Fig. 2 and Supplementary Table S1): *Hexactinellidae, Polychaetae, Demospongia, Ascidiae, Actiniaria*, *Sipunculidae*, *Isopoda, Amphipoda, Echinodermata*. Among those, the following species were analyzed: *Psychropotes longicauda (Holothuroidea: Elasipodida), Molpadia musculus (Holothuroidea: Molpadida), Hymenodora glacialis (Malacostraca: Decapoda), Benthodytes incerta (Holothuroidea: Elasipodida), Calocarides quinqueseriatus (Malacostraca: Decapoda), Munidopsis antonii (Malacostraca: Decapoda), Scotoplanes theeli (Holothuroidea: Elasipodida), Phelliactis callicyclus (Anthozoa: Actiniaria), Atolla sp. (Scyphozoa: Coronatae), Umbellula sp. (Anthozoa: Pennatulacea), Gephyrothuria sp. (Holothuroidea: Molpadida), Psychropotes sp. (Holothuroidea: Elasipodida), Caprella sp. (Malacostraca: Amphipoda), Eunephthea sp. (Anthozoa: Alcyonacea), Peniagone sp. (Holothuroidea: Elasipodida), Corallimorphus sp. (Anthozoa: Corallimorpharia)*, and *Ophiura sp*.,including *Ophiura irrorata (Ophiuroidea: Ophiurida*).

Ethanol extracts (see Methods) from 81 specimens (Fig. 2 and Supplementary Table S1) were screened at different dilutions, using the TNBC cell line BT-20 stably transfected with the TopFlash reporter construct, which expresses a firefly luciferase under the Wnt-dependent TCF-promoter, so that the luminescence activity of the cells reflects the intensity of Wnt signaling^31^, and stimulated by exogenous Wnt3a (provided as Wnt3a-conditioned medium, see Methods). The Wnt3a-conditioned medium is known to be a good substitute for purified Wnt3a for cell culturing purposes and possesses a capability to activate the cascade in the luciferase-based and other cell culture assays and even to support growth of tissue-based organoids *in vitro* ^33^. In addition to the TopFlash firefly luciferase, which measures Wnt3a-induced transcription, the cells were transiently transfected with the CMV-Renilla luciferase reporter a day prior to Wnt stimulation. The Renilla luciferase activity reflects the level of general, Wnt-independent transcription (and thus the well-being of the cells). This dual-luciferase assay allowed us to normalize the values of Wnt-dependent luminescence by the Wnt independent luminescence in the same sample and thus to distinguish between a specific activity of the extracts on the Wnt pathway as opposed to the general effect on cellular transcription and/or general cytotoxicity. The assay used in the study is applicable to track a Wnt-modulating activity working at any level within the cascade, from the upstream ligand-receptor interaction to the downstream transcription factor activity in the nucleus. This allows identifying a broad spectrum of potential inhibitors and modulators during the screening, while their exact molecular target can be deciphered later using other approaches. The specific activity of the cascade in cells treated only with the Wnt3a-conditioned medium (ca. 20-fold over the background), was set to 100%.

This analysis identified 15 extracts from 11 marine invertebrates possessing specific (not affecting general transcription), strong (>2-fold reduction) and significant (p-value by the linear regression test <0.05) Wnt pathway-inhibitory activity (Supplementary Table S2).

Some of these extracts were produced from single specimen of a single species within a larger animal group, such as a decapod crustacean *Calocarides quinqueseriatus* (reduction of Wnt signaling activity to ca. 10% of control at 1:400 dilution), a large unidentified polychaete (ca. 50% at 1:300), or a crab *Munidopsis antonii* (ca. 20% at 1:300) (Supplementary Table S2). In some cases, we were able to delineate a specific body part of the invertebrate, which contained the anti-Wnt activity, such as hepatopancreas, but not muscles, of a decapod crustacean *Calocarides quinqueseriatus* (ca. 10% at 1:400), or skin and muscular sac, but not gonads, of a holothurian *Molpadia musculus* (see further below, Supplementary Table S2).

**Figure 3:**
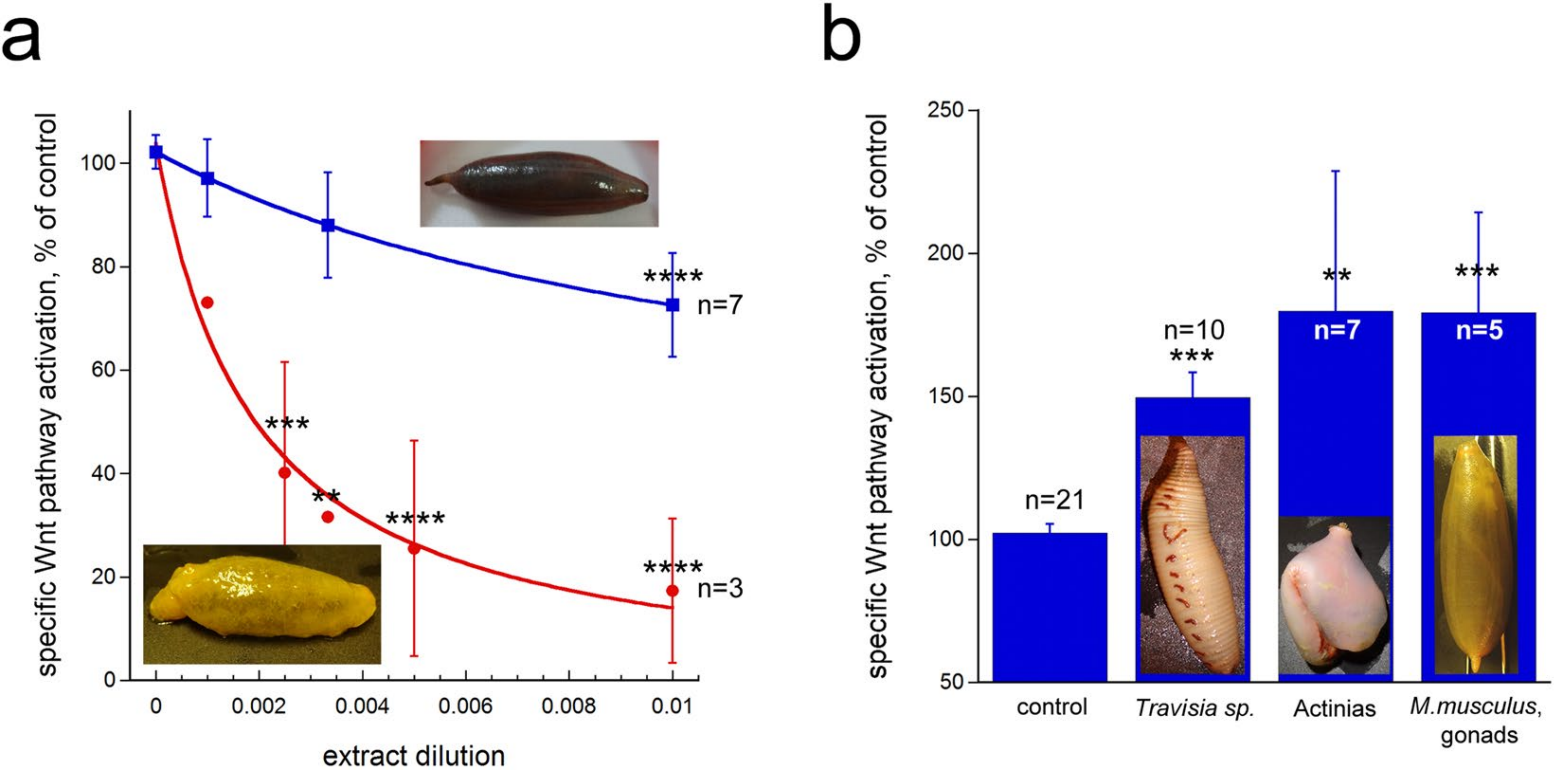
Extracts showing specific Wnt pathway-modulating activities in TNBC cells, across multiple representatives of same invertebrate groups collected at different sites. (**a**) Extracts from holothurians can robustly and specifically inhibit the Wnt pathway. Extracts from *Peniagone sp*. (specimens 59 and 94) are shown in red, and from the skin and muscular sac of *Molpadia musculus* (specimens 4, 37, 54, 86) in blue. The X-axis reflects the dilution of the extract (i.e. the final part of the extract in the culture media, such that dilution of 0.01 corresponds to the 1:100 dilution). The Y-axis shows the activity of the Wnt-dependent luminescence, normalized to the Wnt-independent Renilla luminescence, setting to 100% the normalized luminescence activity of cells treated with the Wnt3a-conditioned medium without any extract added. Curves are fit using the equation: $y={ax}\,-\frac{{bx}}{(c+x)}$, providing *c* as the IC_50_ of the extract-induced inhibition, which is 0.0019 (1:526 dilution) for *Peniagone sp*. and 0.0121 (1:83 dilution) for the skin and muscular sac of *Molpadia musculus*. (**b**) Co-activation of the Wnt pathway can be observed for extracts (all shown for the 1:100 dilution) from several invertebrate groups: polychaete *Travisia sp*. (specimens 7, 17, 53, 64, 65), various Actinias (specimens 16, 44, 55, 78), and gonads of *M.musculus* (specimens 9, 60, 90). Data are presented as mean ± sem. “*” signs highlight experimental points with statistically significant pairwise difference from control (no extract added) as determined by the two-way ANOVA (**a**) and the Student t-test (**b**); p value < 0.05 is indicated as “*”,  <0.01 as “**”,  <0.001 as “***”,  and <0.0001 as “****”. Photographs of the specimens are provided on each panel. “n” stands for the number of experiments.

Much more confidence is provided when different representatives from the same species or of a bigger group, collected at different sites, could be compared. Five invertebrate groups stood out in this regard. Holothurians *Peniagone sp*. and *Molpadia musculus* (skin and muscular sac of the latter), collected at different sites, inhibit the Wnt pathway in a concentration-dependent manner, down to 30% of control at 1:300 for *Peniagone* and to 40% at 1:100 for *Molpadia* (Fig. 3a and Supplementary Table S2). Polychaete *Travisia sp*., various actinias, and gonads of *Molpadia musculus*, in contrast, displayed the ability to co-stimulate the Wnt pathway: activation of the Wnt pathway, induced by addition of Wnt3a, became potentiated by 1.5-2 folds if the extract was added together with the Wnt3a conditioned medium (Fig. 3b and Supplementary Table S2). Given the importance of the Wnt pathway activation in stem cell self-renewal and tissue regeneration^17^, these unusual co-stimulatory activities from marine invertebrates can also be of practical interest^22^ and will require further elaboration of the exact mechanism of action.

**Figure 4:**
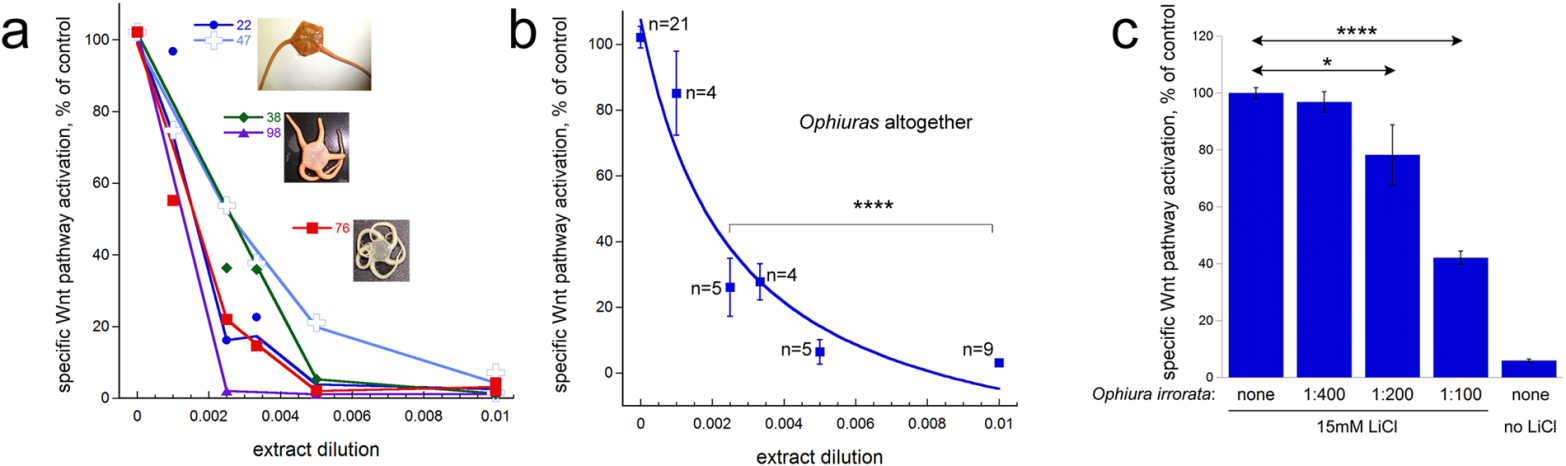
Different ophiuras possess strong and specific anti-Wnt activity in TNBC cells. (**a**,**b**) Extracts from brittle stars collected at station 11 (*Ophiura irrorata*, specimens 22 and 47), 8 (*Ophiura sp*. #1, specimens 38 and 98), and station 9 (*Ophiura sp*. #2, specimen 76), all strongly inhibit the specific Wnt-dependent transcription in a concentration-dependent manner. Curves in (**a**) display representative experiments performed with the indicated specimens. Curve fitting in (**b**) is done as in Fig. 3, with the resulting IC_50_ of 0.0026 (extract dilution of 1:385). (**c**) Extract from *Ophiura irrorata* (#22) also inhibits, in a concentration-dependent manner, Wnt pathway activation induced by 15 mM LiCl. Due to incomplete inhibition, IC_50_ of the inhibition of the LiCl-induced response cannot be adequately calculated. Statistical analysis in (**b,c**) is performed with the 1-way ANOVA. Data presentation is as in Fig. 3. For each dataset in (**c**), n = 3.

The strongest and the most robust effects on the Wnt pathway were seen with the brittle stars (*Ophiura*). Of the five specimens collected at three different stations (station 8 at the depth of 2250 m, station 9 at the depth of 3340 m, and station 11 at the depth of 3140 m, Supplementary Table S1 and Fig. 1) and belonging to 3 different species (Fig. 2) including *Ophiura irrorata*, all showed a near-complete (down to 2–5% of the control at 1:200 dilution) and concentration-dependent inhibition of the Wnt pathway, without noticeable effect on the general cell transcription (Supplementary Table S2 and Fig. 4a,b).

Focusing on *Ophiura irrorata*, we next roughly assessed where in the Wnt signaling pathway the molecular target of the active component(s) of this brittle star could be located. To address this, we compared the ability of the extract to inhibit the Wnt pathway when activated by Wnt3a *vs*. LiCl at 15 mM (a concentration within the standard range for induction of the Wnt signaling in cultured cancer cells^31,34,35^). LiCl inhibits GSK3β, a component of the β-catenin destruction complex, and thus activates the pathway at the cytoplasmic level bypassing the reactions occurring at the plasma membrane and its vicinity^36^. We found that *O. irrorata* could also inhibit this LiCl-induced Wnt pathway activation (Fig. 4c). Curiously, only the highest tested concentration of the extract (the 1:100 dilution) could elicit a clear inhibition of the LiCl-induced pathway activation; further, the induced reduction was only ca. two-fold (Fig. 4c). This contrasts with the near-complete inhibition of the Wnt3a-induced response, already seen with lower concentrations of the extract, and IC_50_ achieved at the ca. 1:400 extract dilution (Fig. 4a,b).

## Discussion

Brittle stars (*Ophiuroidae*) is the largest class of echinoderms, with >2000 species known, of which ca. 400 species reside in the North Pacific^37^. Our finding that specimens from three different *Ophiura* species collected at different locations possess promising activities, inhibiting the Wnt pathway in TNBC cells, hints at the possibility that the same (classes of) anti-Wnt compound(s) may exist in other brittle stars.

Pacific brittle stars have been the source of terpenes and phenylpropanoids (*Ophioplocus* *japonicus* ^38^, steroid sulfates (*Ophiopholis aculeata*, *Ophiura sarsi*, and *Stegophiura brachiactis* ^39^, gangliosides (*Ophiura sarsi* and *Ophiocoma echinata* ^40^ *, Ophiomastrix annulosa* ^41^, and *Ophiocoma scolopendrina* ^42^, and other compounds, some of which are further shared among other echinoderms. Multidimensional fractionation of extracts from *O. irrorata* or other brittle stars, coupled with activity characterization, followed by structure elucidation, are awaiting to identify the molecular nature of the anti-Wnt components we have described here. Subsequent tests *in vitro* and *in vivo* against TNBC (and other Wnt-dependent cancers) will be performed in order to advance these molecules along the path of the anti-cancer drug development^35^. With the recent draft genome sequencing of the brittle star *Ophionereis fasciata* ^43^, potential molecular biology and genetic engineering approaches to characterization and biotechnological applications of the brittle star metabolome will be facilitated.

Extracts from the Persian Gulf *Ophiocoma erinaceus* have been reported to possess anti-proliferative activity against A2780cp cells, and anti-angiogenic activity in the fertilized Ross egg model^44^. Using HeLa cells, anti-migration and pro-apoptotic activities were reported^45^, and the latter were found to be mediated by the polysaccharide fraction of the *O. erinaceus* extracts^46^, while the anti-migration activity – by the saponins^47^. Pacific *O.japonicus* terpenes were also reported to have a certain cytotoxic activity^38^.

However, we must stress here the fundamental difference of our marine natural product-based drug discovery approach from those traditionally applied in the past and also now by many laboratories. We are explicitly not looking for cytotoxic agents. Furthermore, such activities are eliminated during our screening process. Instead, we look for natural compounds targeting one of the hallmarks of cancer – pro-proliferative oncogenic signaling^6^, with a special focus on Wnt signaling responsible for oncogenic transformation, growth and metastasis in a number of tissues, including the breast^22^. Of note, inhibition of Wnt signaling in breast cancer cells causes cell proliferation arrest but not cell death^26,31,35^.

The fact that the *Ophiura irrorata* extract inhibits LiCl and Wnt3a-induced response with different efficiencies suggests that several Wnt-modulatory components exist in brittle stars, acting at different molecular targets within the pathway. One of these targets lies above, and one – below GSK3β. We are particularly interested in the component(s) active against the higher-located molecular target(s). Indeed, inhibitors of the Wnt pathway acting below GSK3β are known to bluntly suppress all the tissue-specific subtypes of the pathway. Thus, not only the oncogenic Wnt signaling, but also physiologic involvements of the pathway relevant e.g. for renewal of epithelial cells in the gastrointestinal tract will be affected producing strong side effects. Instead, it is more promising to selectively target the upper levels of the pathway, where more variability among the signaling subtypes is provided in different tissues and in different contexts (i.e. physiologic *vs*. pathologic)^22,28,48^.

We consider the pipeline of marine-based drug discovery presented here to represent a pioneering approach to deliver novel targeted drug candidates. This pipeline is composed of invertebrate collection during dedicated sea expeditions, specimen extraction, screening using specific assays monitoring oncogenic signaling in cancer cells, and preliminary characterization of the mode of action of the hit extracts within the pathway. As exemplified in this report, such an approach has resulted in identification of promising anti-Wnt and Wnt pathway-modulating activities in *Ophiura* species and also in a number of other invertebrates. Subsequent work will advance these activities towards drug candidates, but also will feed more marine organisms into this pipeline.

## Methods

### Specimen collection and extraction

Invertebrates were collected using the Agassiz Trawl and the epibenthic sledge from depths of 1700–4750 m in the Kuril Basin, Sea of Okhotsk, and adjacent deep-sea area of the Pacific Ocean during the SokhoBio expedition of the research vessel “Akademik M.A. Lavrentiev”^32^ (Supplementary Table S1 and Fig. 1). The specimens were placed in 10 ml 80% ethanol immediately after collection and extracted by storage at 4 °C for 6 months. Each extract sample was derived from a single individual organism of the size average for its species. In case of separate organs or body parts, they were dissected immediately after catch and placed separately into 10 ml 80% ethanol. Before experiments, the ethanol solution, in which the samples had been stored, was collected and centrifuged to remove the debris; the supernatants were concentrated 100-fold in the following way: 1 ml of the extract was put in a 1.5 ml tube and evaporated under vacuum at 30 °C using the Eppendorf concentrator 5301. The dry pellet was then re-dissolved in 10 μl DMSO and stored at −20 °C.

### Ethics statement

Invertebrate sample collection was performed by the standards approved by the Ministry of Education and Science (Russia), and Federal Ministry of Education and Research (Germany). All efforts were made to minimize suffering.

### Wnt3a-conditioned medium

The Wnt3a-conditioned medium was obtained by culturing mouse L-cells stably transfected with Wnt3a (A.T.C.C. catalogue number CRL-2647) with the initial confluency of 10–20% for 4 days in DMEM (Dulbecco’s modified Eagle’s medium) supplemented with 10% FBS (fetal bovine serum; HyClone), as described^49^. Before utilization, the medium was centrifugated at 4000 g for 5 min, filter-sterilized (0.22 µm), tested for its ability to activate Wnt-dependent luminescence over the background level in BT-20-Tf cells (see below), aliquoted and stored at −20 °C.

### Luciferase-based assay of the Wnt-dependent transcriptional activity

BT-20 (A.T.C.C. catalogue number HTB-19), a triple-negative human mammary carcinoma line cells stably transfected with the M50 Super 8 × TopFlash plasmid^31^ were used to analyze the Wnt modulatory activities of extracts from marine invertebrates. The assay was performed in white tissue-culture-treated 96-well plates (Greiner). The BT-20-Tf cells were seeded in 100 μl of DMEM containing 10% FBS at ca. 10000 cells/well, incubated overnight at 37 °C, 5% CO_2_ and subsequently stimulated by 50 μl of the Wnt3a-conditioned medium or LiCl (to 15 mM) in the presence of the invertebrate extracts to the final concentration of DMSO of 0.25%, or DMSO alone, for 12–24 h. If indicated, the cells were additionally transiently transfected by the pCMV-RL plasmid (purified with Quiagen Plasmid Midi Kit 12145) for constitutive expression of *Renilla* luciferase^50^ using XtremeGENE 9 reagent (Roche) according to the manufacturer’s protocol 16–24 h prior to treatment with the Wnt3a conditioned medium and the extracts. The medium was subsequently removed and 15 μl of the lysis buffer (25 mM glycylglycine, pH 7.8, 1% Triton X-100, 15 mM MgSO_4_, 4 mM EGTA, 1 mM DTT) were pipetted into each well. After incubation for 5 min at room temperature, the 96-well plate was analyzed using the Victor3 Multilabel Counter (PerkinElmer) or Cytation 5 (Biotek) with the two-channel dispensing unit primed with the buffer solutions for separate activity measurements of firefly and *Renilla* luciferase, prepared as described^51^. The final volumes dispensed per well were 50 μl of firefly and 50 μl of *Renilla* solutions.

### Electronic supplementary material

The online version of this article contains supplementary material (Supplementary Tables S1 and S2), which is available to authorized users.

### Data availability statement

All data generated or analysed during this study are included in this published article and its Supplementary Information files.

## Electronic supplementary material

Supplementary Information 

Supplementary Table S1

Supplementary Table S2

## References

[1] Newman DJ, Cragg GM (2007). Natural products as sources of new drugs over the last 25 years. J Nat Prod.

[2] Kong DX, Jiang YY, Zhang HY (2010). Marine natural products as sources of novel scaffolds: achievement and concern. Drug Discov Today.

[3] Martins A, Vieira H, Gaspar H, Santos S (2014). Marketed marine natural products in the pharmaceutical and cosmeceutical industries: tips for success. Mar Drugs.

[4] Katzung, B., Masters, S. & Trevor, A. *Basic and Clinical Pharmacology 12/E*, (McGraw-Hill Companies, Inc., 2011).

[5] Newman DJ, Cragg GM (2014). Marine-sourced anti-cancer and cancer pain control agents in clinical and late preclinical development. Mar Drugs.

[6] Hanahan D, Weinberg RA (2000). The hallmarks of cancer. Cell.

[7] Rudin CM (2012). Vismodegib. Clin Cancer Res.

[8] Polyak K, Metzger Filho O (2012). SnapShot: breast cancer. Cancer Cell.

[9] Jordan VC (2003). Tamoxifen: a most unlikely pioneering medicine. Nat Rev Drug Discov.

[10] Arteaga CL (2012). Treatment of HER2-positive breast cancer: current status and future perspectives. Nat Rev Clin Oncol.

[11] Anders CK, Carey LA (2009). Biology, metastatic patterns, and treatment of patients with triple-negative breast cancer. Clin Breast Cancer.

[12] Youlden DR (2012). The descriptive epidemiology of female breast cancer: an international comparison of screening, incidence, survival and mortality. Cancer Epidemiol.

[13] King TD, Suto MJ, Li Y (2012). The Wnt/beta-catenin signaling pathway: a potential therapeutic target in the treatment of triple negative breast cancer. J Cell Biochem.

[14] Pohl SG (2017). Wnt signaling in triple-negative breast cancer. Oncogenesis.

[15] Rangel MC (2016). Developmental signaling pathways regulating mammary stem cells and contributing to the etiology of triple-negative breast cancer. Breast Cancer Res Treat.

[16] Logan CY, Nusse R (2004). The Wnt signaling pathway in development and disease. Annu Rev Cell Dev Biol.

[17] Reya T, Clevers H (2005). Wnt signalling in stem cells and cancer. Nature.

[18] Giles RH, van Es JH, Clevers H (2003). Caught up in a Wnt storm: Wnt signaling in cancer. Biochimica et Biophysica Acta.

[19] Turashvili G, Bouchal J, Burkadze G, Kolar Z (2006). Wnt signaling pathway in mammary gland development and carcinogenesis. Pathobiology.

[20] Xu J, Prosperi JR, Choudhury N, Olopade OI, Goss KH (2015). beta-Catenin is required for the tumorigenic behavior of triple-negative breast cancer cells. PLoS One.

[21] Lehmann BD (2011). Identification of human triple-negative breast cancer subtypes and preclinical models for selection of targeted therapies. J Clin Invest.

[22] Blagodatski A, Poteryaev D, Katanaev V (2014). Targeting the Wnt pathways for therapies. Molecular and Cellular Therapies.

[23] MacDonald BT, Tamai K, He X (2009). Wnt/beta-catenin signaling: components, mechanisms, and diseases. Dev Cell.

[24] Katanaev, V., Koval, A. & Blagodatski, A. Wnt7a polypeptides and their use. In *European patent application 20140718, filed 18.07.2014* (2014).

[25] Liu CC, Prior J, Piwnica-Worms D, Bu G (2010). LRP6 overexpression defines a class of breast cancer subtype and is a target for therapy. Proc Natl Acad Sci USA.

[26] Yang L (2011). FZD7 has a critical role in cell proliferation in triple negative breast cancer. Oncogene.

[27] Gurney, A. *et al*. Wnt pathway inhibition via the targeting of Frizzled receptors results in decreased growth and tumorigenicity of human tumors. *Proc Natl Acad Sci USA* (2012). 10.1073/pnas.1120068109 PMC3406803 22753465

[28] Koval A, Katanaev VL (2012). Platforms for high-throughput screening of Wnt/Frizzled antagonists. Drug Discov Today.

[29] Huang SM (2009). Tankyrase inhibition stabilizes axin and antagonizes Wnt signalling. Nature.

[30] Janda CY, Waghray D, Levin AM, Thomas C, Garcia KC (2012). Structural basis of Wnt recognition by Frizzled. Science.

[31] Koval AV (2014). Anti-leprosy drug clofazimine inhibits growth of triple-negative breast cancer cells via inhibition of canonical Wnt signaling. Biochem Pharmacol.

[32] Malyutina, M., Brandt, A. & Ivin, V. V. The Russian-German deep-sea expedition SokhoBio (Sea of Okhotsk Biodiversity Studies) to the Kurile Basin of the Sea of Okhotsk on board of the R/V Akademik M.A.Lavrentyev. 71st Cruise, July 6th - August 6th, 2015. The Cruise Report. 1–103 (Report submitted to: Ministry of Education and Science (Russia) and Federal Ministry of Education and Research (Germany), (2015).

[33] Broutier L (2016). Culture and establishment of self-renewing human and mouse adult liver and pancreas 3D organoids and their genetic manipulation. Nat Protoc.

[34] Sievers S, Fritzsch C, Grzegorczyk M, Kuhnen C, Muller O (2006). Absolute beta-catenin concentrations in Wnt pathway-stimulated and non-stimulated cells. Biomarkers.

[35] Koval A, Ahmed K, Katanaev VL (2016). Inhibition of Wnt signalling and breast tumour growth by the multi-purpose drug suramin through suppression of heterotrimeric G proteins and Wnt endocytosis. Biochem J.

[36] Klein PS, Melton DA (1996). A molecular mechanism for the effect of lithium on development. Proc Natl Acad Sci U S A.

[37] Stohr S, O’Hara TD, Thuy B (2012). Global diversity of brittle stars (Echinodermata: Ophiuroidea). PLoS One.

[38] Wang WH (2004). Bioactive Metabolites from the Brittle Star Ophioplocus japonicus. Natural Product Sciences.

[39] Fedorov SN, Levina EV, Kalinovsky AI, Dmitrenok PS, Stonik VA (1994). Sulfated Steroids from Pacific Brittle Stars Ophiopholis aculeata, Ophiura sarsi, and Stegophiura brachiactis. Journal of Natural Products.

[40] Smirnova GP, Chekareva NV, Kochetkov NK (1986). Gangliosides of Ophiura Ophiura-Sarsi. Bioorganicheskaya Khimiya.

[41] Chekareva NV, Smirnova GP, Kochetkov NK (1991). Gangliosides from 2 Species of Ophiura, Ophiocoma-Echinata and Ophiomastix-Annulosa Clark. Bioorganicheskaya Khimiya.

[42] Inagaki M, Shibai M, Isobe R, Higuchi R (2001). Constituents of ophiuroidea. 1. Isolation and structure of three ganglioside molecular species from the brittle star Ophiocoma scolopendrina. Chem Pharm Bull (Tokyo).

[43] Long KA, Nossa CW, Sewell MA, Putnam NH, Ryan JF (2016). Low coverage sequencing of three echinoderm genomes: the brittle star Ophionereis fasciata, the sea star Patiriella regularis, and the sea cucumber Australostichopus mollis. Gigascience.

[44] Baharara J, Amini E, Mousavi M (2015). The anti-proliferative and anti-angiogenic effect of the methanol extract from brittle star. Rep Biochem Mol Biol.

[45] Baharara J, Amini E, Namvar F (2016). Evaluation of the Anti-proliferative Effects of Ophiocoma erinaceus Methanol Extract Against Human Cervical Cancer Cells. Avicenna J Med Biotechnol.

[46] Baharara J, Amini E (2015). The Potential of Brittle Star Extracted Polysaccharide in Promoting Apoptosis via Intrinsic Signaling Pathway. Avicenna J Med Biotechnol.

[47] Amini E, Nabiuni M, Baharara J, Parivar K, Asili J (2015). Metastatic Inhibitory and Radical Scavenging Efficacies of Saponins Extracted from the Brittle Star (Ophiocoma erinaceus). Asian Pac J Cancer Prev.

[48] Katanaev VL (2014). Prospects of Targeting Wnt Signaling in Cancer. J Pharmacol Toxicol Res.

[49] Willert K (2003). Wnt proteins are lipid-modified and can act as stem cell growth factors. Nature.

[50] Mosimann C, Hausmann G, Basler K (2009). The role of Parafibromin/Hyrax as a nuclear Gli/Ci-interacting protein in Hedgehog target gene control. Mech Dev.

[51] Dyer BW, Ferrer FA, Klinedinst DK, Rodriguez R (2000). A noncommercial dual luciferase enzyme assay system for reporter gene analysis. Anal Biochem.

