# Supplementary material for: High-throughput targeted screening in triple-negative breast cancer cells identifies Wnt-inhibiting activities in Pacific brittle stars
Source: Sci Rep. 2017 Sep 20;7:11964. doi: 10.1038/s41598-017-12232-7 (PMC5607299; doi:10.1038/s41598-017-12232-7)
Supplement: Supplementary file 1 — Supplementary Information [file 41598_2017_12232_MOESM1_ESM.pdf]

High-throughput targeted screening in triple-negative breast cancer cells identifies Wnt-inhibiting activities in Pacific brittle stars

Artem Blagodatski, Vsevolod Cherepanov, Alexey Koval, Vladimir I. Kharlamenko, Yuri S. Khotimchenko, and Vladimir L. Katanaev

Supplementary Tables S1 and S2
