# Supplementary material for: High-throughput targeted screening in triple-negative breast cancer cells identifies Wnt-inhibiting activities in Pacific brittle stars
Source: Sci Rep. 2017 Sep 20;7:11964. doi: 10.1038/s41598-017-12232-7 (PMC5607299; doi:10.1038/s41598-017-12232-7)
Supplement: Supplementary file 2 — Supplementary Table S1 [file 41598_2017_12232_MOESM2_ESM.doc]

**Supplementary Table 1. Details on invertebrate sample collection during the SokhoBio expedition**

| **Taxonomy** | **Species** |  |  | **Sample number(s)** | **Station** | **Depth** | **Trawl** | **Part of animal, comments** |
| --- | --- | --- | --- | --- | --- | --- | --- | --- |
| **Porifera (Phylum)** |  |  |  |  |  |  |  |  |
| **Demospongiae (Class)** |  |  |  |  |  |  |  |  |
|  | Demosponge #1 | |  | 15 | Station 2 | 3352 м | Trawl 2-9 | piece of sponge |
|  | Demosponge #2 | |  | 58;70;14 | Station 2 | 3352 м | Trawl 2-9 | piece of sponge |
|  | Demosponge #3 | |  | 21;84 | Station 3 | 3363 м | Trawl 3-7 | piece of sponge |
|  | Demosponge #4 | |  | 50 | Station 6 | 3347 м | Trawl 6-8 | piece of sponge |
|  | Demosponge #5 | |  | 43 | Station 6 | 3347 м | Trawl 6-8 | piece of sponge |
|  | Demosponge #7 | |  | 31;33;99 | Station 7 | 3300 м | Trawl 7-12 | piece of sponge |
|  | Demosponge #8 | |  | 30;68 | Station 8 | 2250 м | Trawl 8-6 | piece of sponge |
|  | Demosponge #9 | |  | 69 | Station 8 | 2250 м | Trawl 8-6 | piece of sponge |
|  | Demosponge #10 | |  | 13 | Station 2 | 3352 м | Trawl 2-9 | piece of sponge |
|  | Demosponge #10 | |  | 79;92 | Station 4 | 3366 м | Trawl 4-2 | piece of sponge |
| **Hexactinellida (Class)** |  |  |  |  |  |  |  |  |
|  | *Hexactinellid* sponge #1 | | | 74 | Station 1 | 3305 m | Trawl 1-11 | piece of sponge |
|  | *Hexactinellid* sponge #2 | | | 34 | Station2 | 3352 м | Trawl 2-9 | piece of sponge |
|  | *Hexactinellid* sponge #3 | | | 39 | Station2 | 3352 м | Trawl 2-9 | piece of dead sponge |
|  | *Hexactinellid* sponge #4 | | | 71 | Station2 | 3352 м | Trawl 2-9 | piece of sponge |
|  | *Hyalonema sp.* 1 | |  | 82 | Station2 | 3352 m | Sledge 2-8 |  |
|  | *Hyalonema sp.* 1 | |  | 88 | Station2 | 3352 m | Trawl 2-9 |  |
|  | *Hyalonema sp.* 2 | |  | 57;83;89 | Station 7 | 3300 м | Trawl 7-11 | piece of sponge |
| **Cnidaria (Phylum)** |  |  |  |  |  |  |  |  |
| **Scyphozoa (Class)** |  |  |  |  |  |  |  |  |
| **Coronatae (Order)** |  |  |  |  |  |  |  |  |
|  | *Atolla wyville* | |  | 3;5;6 | 3305 m | Station 1 | Trawl 1-10 |  |
| **Anthozoa (Class)** |  |  |  |  |  |  |  |  |
| **Actiniaria (Order)** |  |  |  |  |  |  |  |  |
|  | *Phelliactis callicyclus* | | | 16 | 3305 m | Station 1 | Trawl 1-11 | body wall |
|  | *Phelliactis callicyclus* | | | 44;12 | 3352 m | Station 2 | Trawl 2-9 | body wall |
|  | *Actinia* |  |  | 55;78 | 1700 м | Station 5 | Trawl 5-9 | body wall |
| **Corallimorpharia (Order)** |  |  |  |  |  |  |  |  |
|  | *Corallimorpharia sp.* 1 | | | 25 | 3340 м | Station9 | Trawl 9-10 | whole animal |
| **Alcyonacea (Order)** |  |  |  |  |  |  |  |  |
|  | *Eunephthya sp.* | |  | 91 | 2250 м | Station 8 | Trawl 8-6 | whole animal |
|  | *Gorgoniidae sp.* 1 | |  | 11 |  |  |  | whole animal |
| **Pennatulacea (Order)** |  |  |  |  |  |  |  |  |
|  | *Umbellula  sp.* | |  | 8;35;87 | 4750 м | Station 10 | Sledge 10-6 | polyps |
| **Annelida (Phylum)** |  |  |  |  |  |  |  |  |
| **Polychaeta (Class)** |  |  |  |  |  |  |  |  |
| **Phyllodocida (Order)** |  |  |  |  |  |  |  |  |
|  | *Laetmonice wyvillei* | | | 93 | 3340 м | Station9 | Trawl 9-10 | body wall |
| **Scolecida (Infraclass)** |  |  |  |  |  |  |  |  |
|  | *Travisia  sp.*1 | |  | 7;17;64 | 3352 м | Station 2 | Trawl 2-9 | body wall |
|  | *Travisia  sp.*2 | |  | 53;65 | 1700 м | Station 5 | Trawl 5-9 | body wall |
| **Echiuroidea (Order)** |  |  |  |  |  |  |  |  |
|  | *Pseudoikedella achaeta* | | | 1;73 | 3363 м | Station 3 | Trawl 3-7 | body wall |
| **Sipuncula (Phylum)** |  |  |  |  |  |  |  |  |
| **Sipunculidea (Class)** |  |  |  |  |  |  |  |  |
|  | *Golfingia margaritacea* | | | 40;81 | 3140 м | Station 11 | Trawl 11-7 | body wall |
| **Arthropoda (Phylum)** |  |  |  |  |  |  |  |  |
| **Malacostraca (Class)** |  |  |  |  |  |  |  |  |
| **Decapoda (Order)** |  |  |  |  |  |  |  |  |
|  | *Munidopsis antonii* | | | 96 | 3140 м | Station 11 | Trawl 11-7 | hepatopancreas |
|  | *Calocarides quinqueseriatus* | | | 97 | 1700 м | Station 5 | Trawl 5-10 | hepatopancreas |
|  | *Calocarides quinqueseriatus* | | | 46 | 1700 м | Station 5 | Trawl 5-10 | muscles |
|  | *Hymenodora glacialis* | | | 48;52;62;80 | 3305 m | Station 1 | Trawl 1-11 | muscles |
| **Amphipoda (Order)** |  |  |  |  |  |  |  |  |
|  | *Caprella sp.* | |  | 67 | 2250 м | Station 8 | Trawl 8-6 | whole animal |
| **Isopoda (Order)** |  |  |  |  |  |  |  |  |
|  | *Tecticeps sp.* | |  | 85 | 2250 м | Station 8 | Trawl 8-6 | whole animal |
| **Echinodermata (Phylum)** |  |  |  |  |  |  |  |  |
| **Ophiuroidea (Class)** |  |  |  |  |  |  |  |  |
|  | *Ophiura sp.* #1 | |  | 38;63;98 | 2250 м | Station 8 | Trawl 8-6 | rays |
|  | *Ophiura sp.* #2 | |  | 28;76 | 3340 м | Station9 | Trawl 9-10 | rays |
|  | *Ophiura irrorata* | |  | 22;47 | 3140 м | Station 11 | Trawl 11-7 | digestive glands |
| **Echinoidea (Class)** |  |  |  |  |  |  |  |  |
|  | *Cystechinus loveni* | | | 27;45;56 | 3340 м | Station9 | Trawl 9-9 | gonads |
| **Holothuroidea (Class)** |  |  |  |  |  |  |  |  |
|  | *Peniagone sp.* | |  | 2;20;26 | 4750 м | Station 10 | Trawl 10-7 | body wall |
|  | *Peniagone inserta* | |  | 59;94 | 3305 m | Station 1 | Trawl 1-11 | body wall |
|  | *Scotoplanes theeli* | | | 18;36 | 3340 м | Station9 | Trawl 9-9 | body wall |
|  | *Scotoplanes theeli* | | | 19;29;32 | 3340 м | Station9 | Trawl 9-9 | gonads |
|  | *Benthodytes incerta* | | | 23;66;72 | 3305 m | Station 1 | Trawl 1-11 | body wall |
|  | *Psychropotes sp.* | |  | 24 | 3300 м | Station 7 | Trawl 7-11 | body wall |
|  | *Gephyrothuria sp.* | |  | 51;95 | 4750 м | Station 10 | Trawl 10-7 | body wall |
|  | *Molpadia musculus* | | | 4;49;54 | 3305 m | Station 1 | Trawl 1-11 | body wall |
|  | *Molpadia musculus* | | | 61 | 3363 м | Station 3 | Trawl 3-7 | body wall |
|  | *Molpadia musculus* | | | 9;60 | 3366 м | Station 4 | Trawl 4-2 | gonads |
|  | *Molpadia musculus* | | | 37;86 | 4750 м | Station 10 | Trawl 10-7 | body wall |
|  | *Molpadia musculus* | | | 90 | 4750 м | Station 10 | Trawl 10-7 | gonads |
|  | *Molpadia musculus* | | | 41;42;77 | 3140 м | Station 11 | Trawl 11-7 | body wall |
