# Supplementary material for: High-throughput targeted screening in triple-negative breast cancer cells identifies Wnt-inhibiting activities in Pacific brittle stars
Source: Sci Rep. 2017 Sep 20;7:11964. doi: 10.1038/s41598-017-12232-7 (PMC5607299; doi:10.1038/s41598-017-12232-7)
Supplement: Supplementary file 3 — Supplementary Table S2 [file 41598_2017_12232_MOESM3_ESM.doc]

**Supplementary Table 2. Results of the testing of invertebrate extracts on the Wnt-specific transcriptional response (TopFlash) and general cell transcription (*Renilla*).**

**Linear regression analysis (forcing to zero) is performed with the JMP 10 software to test significance of the difference between the effect of the extract on the TopFlash firefly luciferase and the CMV-*Renilla* luciferase expression.**

| Specimen # | Specimen taxonomy and body part (where applicable) | extract dilution | TopFlash luciferase | *Renilla* luciferase | TofFlash/ *Renilla*, % | linear regression; p-value where significant | linear regression for group; p-value where significant | comments |
| --- | --- | --- | --- | --- | --- | --- | --- | --- |
| 3 | Jellyfish, *Atolla sp.* | 1:100 | 86.20 | 62.55 | 137.82 | 0.0074 |  |  |
|  |  | 1:100 | 78.42 | 70.65 | 110.99 |  |  |  |
|  |  | 1:300 | 95.45 | 84.69 | 112.70 |  |  |  |
|  |  | 1:300 | 103.93 | 88.52 | 117.40 |  |  |  |
|  |  | 1:1000 | 110.46 | 95.90 | 115.17 |  |  |  |
|  |  | 1:1000 | 102.70 | 95.45 | 107.60 |  |  |  |
| 5 | Jellyfish, *Atolla sp.* | 1:100 | 69.65 | 78.99 | 88.17 |  |  |  |
|  |  | 1:300 | 84.75 | 96.85 | 87.50 |  |  |  |
|  |  | 1:1000 | 88.30 | 78.97 | 111.82 |  |  |  |
| 6 | Jellyfish, *Atolla sp.* | 1:100 | 107.73 | 86.38 | 124.73 | 0.0534 |  |  |
|  |  | 1:100 | 106.27 | 93.13 | 114.12 |  |  |  |
|  |  | 1:300 | 99.62 | 101.53 | 98.12 |  |  |  |
|  |  | 1:300 | 97.33 | 94.01 | 103.53 |  |  |  |
|  |  | 1:1000 | 110.04 | 118.77 | 92.65 |  |  |  |
|  |  | 1:1000 | 126.79 | 100.72 | 125.88 |  |  |  |
| 2 | Holothurian, Peniagone-like, skin and muscular sac | 1:100 | 109.61 | 92.92 | 117.96 | 0.0057 |  |  |
|  |  | 1:100 | 106.56 | 88.29 | 120.69 |  |  |  |
|  |  | 1:300 | 102.27 | 92.59 | 110.45 |  |  |  |
|  |  | 1:300 | 114.61 | 98.33 | 116.55 |  |  |  |
|  |  | 1:1000 | 102.27 | 92.59 | 110.45 |  |  |  |
|  |  | 1:1000 | 114.61 | 98.33 | 116.55 |  |  |  |
| 20 | Holothurian, Peniagone-like, skin and muscular sac | 1:100 | 67.49 | 115.42 | 58.47 |  |  |  |
|  |  | 1:300 | 75.23 | 114.27 | 65.83 |  |  |  |
|  |  | 1:1000 | 93.35 | 98.04 | 95.22 |  |  |  |
| **59** | **Holothurian, *Peniagone sp.*** | 1:100 | 81.92 | 181.16 | **45.22** | **0.0228** | **0.0002** | **Peniagone sp. altogether** |
|  |  | 1:200 | 64.54 | 139.12 | **46.39** |  |  |  |
|  |  | 1:400 | 69.28 | 112.52 | 61.57 |  |  |  |
| **94** | **Holothurian, *Peniagone sp.*** | 1:100 | 3.77 | 72.51 | **5.19** | **0.0011** |  |  |
|  |  | 1:300 | 30.53 | 96.58 | **31.61** |  |  |  |
|  |  | 1:1000 | 54.12 | 74.08 | 73.07 |  |  |  |
|  |  | 1:100 | 1.45 | 90.99 | **1.59** |  |  |  |
|  |  | 1:200 | 5.05 | 108.88 | **4.64** |  |  |  |
|  |  | 1:400 | 24.09 | 128.69 | **18.72** |  |  |  |
| **4** | **Holothurian, *Molpadia musculus*, skin and muscular sac** | 1:100 | 44.93 | 104.35 | **43.06** | **0.0413** | **0.0019** | **skin and muscular sac altogether** |
|  |  | 1:300 | 62.10 | 120.74 | 51.43 |  |  |  |
|  |  | 1:1000 | 71.27 | 101.14 | 70.47 |  |  |  |
| **9** | **Holothurian, *Molpadia musculus*, gonads** | 1:100 | 120.34 | 79.20 | **151.93** | **0.0321** | **0.0066** | **gonads altogether** |
|  |  | 1:100 | 103.93 | 85.76 | 121.18 |  |  |  |
|  |  | 1:300 | 92.68 | 114.63 | 80.85 |  |  |  |
|  |  | 1:300 | 109.97 | 93.11 | 118.10 |  |  |  |
|  |  | 1:1000 | 106.97 | 120.42 | 88.83 |  |  |  |
|  |  | 1:1000 | 94.94 | 101.03 | 93.97 |  |  |  |
| **37** | **Holothurian, *Molpadia musculus*, skin and muscular sac** | 1:100 | 41.01 | 99.35 | **41.28** | **0.0143** |  |  |
|  |  | 1:300 | 61.20 | 99.30 | 61.63 |  |  |  |
|  |  | 1:1000 | 87.68 | 107.49 | 81.57 |  |  |  |
|  |  | 1:100 | 62.50 | 116.91 | 53.46 |  |  |  |
|  |  | 1:200 | 106.87 | 114.32 | 93.49 |  |  |  |
|  |  | 1:400 | 139.81 | 107.86 | 129.63 |  |  |  |
| 54 | Holothurian, *Molpadia musculus*, skin and muscular sac | 1:100 | 64.68 | 88.32 | 73.23 | **0.0091** |  |  |
|  |  | 1:100 | 62.63 | 96.69 | 64.77 |  |  |  |
|  |  | 1:300 | 94.91 | 91.93 | 103.24 |  |  |  |
|  |  | 1:300 | 109.23 | 98.52 | 110.87 |  |  |  |
|  |  | 1:1000 | 108.90 | 109.96 | 99.04 |  |  |  |
|  |  | 1:1000 | 98.44 | 99.08 | 99.36 |  |  |  |
| 60 | Holothurian, *Molpadia musculus*, gonads | 1:100 | 51.98 | 52.22 | 99.53 |  |  |  |
|  |  | 1:300 | 46.59 | 63.50 | 73.36 |  |  |  |
|  |  | 1:1000 | 49.45 | 58.34 | 84.77 |  |  |  |
| 86 | Holothurian, *Molpadia musculus*, skin and muscular sac | 1:100 | 107.77 | 86.61 | 124.43 | 0.5454 |  |  |
|  |  | 1:100 | 80.82 | 92.93 | 86.98 |  |  |  |
|  |  | 1:300 | 96.73 | 98.03 | 98.67 |  |  |  |
|  |  | 1:300 | 97.76 | 95.39 | 102.48 |  |  |  |
|  |  | 1:1000 | 106.11 | 90.87 | 116.77 |  |  |  |
|  |  | 1:1000 | 114.47 | 99.07 | 115.55 |  |  |  |
| **90** | **Holothurian, *Molpadia musculus*, gonads** | 1:100 | 131.64 | 47.92 | **274.73** | **0.0049** |  |  |
|  |  | 1:100 | 158.74 | 63.79 | **248.87** |  |  |  |
|  |  | 1:300 | 76.25 | 99.87 | 76.35 |  |  |  |
|  |  | 1:300 | 67.41 | 105.06 | 64.17 |  |  |  |
|  |  | 1:1000 | 109.70 | 107.56 | 101.99 |  |  |  |
|  |  | 1:1000 | 97.70 | 93.96 | 103.98 |  |  |  |
| 42 | Holothurian, *Molpadia sp*., skin and muscular sac | 1:100 | 59.66 | 109.02 | 54.73 | 0.1167 |  |  |
|  |  | 1:300 | 65.56 | 132.61 | 49.44 |  |  |  |
|  |  | 1:1000 | 68.52 | 118.65 | 57.75 |  |  |  |
| 61 | Holothurian, *Molpadia sp*. | 1:100 | 107.07 | 82.13 | 130.37 |  |  |  |
|  |  | 1:100 | 99.09 | 91.30 | 108.53 |  |  |  |
|  |  | 1:300 | 97.55 | 111.03 | 87.86 |  |  |  |
|  |  | 1:300 | 90.37 | 99.87 | 90.49 |  |  |  |
|  |  | 1:1000 | 95.92 | 93.39 | 102.71 |  |  |  |
|  |  | 1:1000 | 111.26 | 94.67 | 117.52 |  |  |  |
| 23 | Holothurian, *Benthodytes incerta*, skin and muscular sac | 1:100 | 101.16 | 86.26 | 117.27 |  |  |  |
|  |  | 1:100 | 117.82 | 89.30 | 131.93 |  |  |  |
|  |  | 1:300 | 94.04 | 93.92 | 100.13 |  |  |  |
|  |  | 1:300 | 101.21 | 101.45 | 99.77 |  |  |  |
| 66 | Holothurian, *Benthodytes incerta*, skin and muscular sac | 1:100 | 97.56 | 93.11 | 104.78 |  |  |  |
|  |  | 1:100 | 111.91 | 101.66 | 110.08 |  |  |  |
|  |  | 1:300 | 100.77 | 94.80 | 106.30 |  |  |  |
|  |  | 1:300 | 93.31 | 105.05 | 88.82 |  |  |  |
|  |  | 1:1000 | 111.04 | 99.48 | 111.62 |  |  |  |
|  |  | 1:1000 | 110.12 | 127.46 | 86.40 |  |  |  |
| 72 | Holothurian, *Benthodytes incerta*, skin and muscular sac | 1:100 | 94.01 | 87.01 | 108.04 |  |  |  |
|  |  | 1:100 | 105.46 | 95.29 | 110.67 |  |  |  |
|  |  | 1:300 | 90.06 | 91.89 | 98.01 |  |  |  |
|  |  | 1:300 | 101.64 | 97.71 | 104.02 |  |  |  |
| 75 | Holothurian, *Benthodytes incerta*, skin and muscular sac | 1:100 | 108.28 | 92.13 | 117.52 |  |  |  |
|  |  | 1:100 | 139.73 | 89.93 | 155.37 |  |  |  |
|  |  | 1:300 | 98.73 | 100.81 | 97.94 |  |  |  |
|  |  | 1:300 | 102.23 | 100.85 | 101.37 |  |  |  |
|  |  | 1:1000 | 110.61 | 96.53 | 114.58 |  |  |  |
|  |  | 1:1000 | 110.89 | 97.28 | 113.99 |  |  |  |
| 24 | Holothurian, *Psychropotes sp*. | 1:100 | 64.95 | 107.12 | *60.63* | 0.028 |  |  |
|  |  | 1:300 | 79.28 | 122.63 | 64.65 |  |  |  |
|  |  | 1:1000 | 103.29 | 110.98 | 93.08 |  |  |  |
| 51 | Holothurian, *Gephyrothuria sp*. | 1:100 | 120.72 | 113.30 | 106.56 |  |  |  |
|  |  | 1:100 | 138.84 | 102.63 | 135.28 |  |  |  |
|  |  | 1:300 | 126.04 | 124.09 | 101.57 |  |  |  |
|  |  | 1:300 | 122.97 | 131.27 | 93.68 |  |  |  |
| 95 | Holothurian, *Gephyrothuria sp*. | 1:100 | 95.16 | 96.31 | 98.80 |  |  |  |
|  |  | 1:100 | 83.62 | 85.15 | 98.20 |  |  |  |
|  |  | 1:300 | 98.24 | 94.69 | 103.75 |  |  |  |
|  |  | 1:300 | 94.98 | 97.53 | 97.38 |  |  |  |
| 7 | Polychaete, *Travisia sp*. | 1:100 | 126.76 | 106.99 | 118.47 | **0.0253** | **<0.0001** | ***Travisia* altogether** |
|  |  | 1:100 | 132.57 | 100.73 | 131.61 |  |  |  |
|  |  | 1:300 | 90.12 | 99.19 | 90.86 |  |  |  |
|  |  | 1:300 | 101.26 | 103.00 | 98.31 |  |  |  |
|  |  | 1:1000 | 104.00 | 108.42 | 95.92 |  |  |  |
|  |  | 1:1000 | 110.95 | 95.39 | 116.31 |  |  |  |
| 17 | Polychaete, *Travisia sp*. | 1:100 | 106.12 | 105.35 | 100.74 | 0.0748 |  |  |
|  |  | 1:100 | 124.16 | 88.11 | 140.91 |  |  |  |
|  |  | 1:300 | 91.50 | 92.24 | 99.20 |  |  |  |
|  |  | 1:300 | 102.14 | 75.69 | 134.94 |  |  |  |
|  |  | 1:1000 | 107.23 | 105.83 | 101.32 |  |  |  |
|  |  | 1:1000 | 95.75 | 95.21 | 100.56 |  |  |  |
| **53** | **Polychaete, *Travisia sp*.** | 1:100 | 162.67 | 87.75 | **185.37** | **0.0066** |  |  |
|  |  | 1:100 | 167.78 | 112.69 | 148.89 |  |  |  |
|  |  | 1:300 | 120.57 | 101.54 | 118.74 |  |  |  |
|  |  | 1:300 | 106.30 | 110.07 | 96.58 |  |  |  |
| **64** | **Polychaete, *Travisia sp*.** | 1:100 | 146.02 | 87.93 | **166.06** | **0.0002** |  |  |
|  |  | 1:100 | 137.61 | 88.78 | **155.00** |  |  |  |
|  |  | 1:300 | 95.91 | 92.05 | 104.20 |  |  |  |
|  |  | 1:300 | 103.94 | 99.87 | 104.08 |  |  |  |
|  |  | 1:1000 | 109.95 | 101.06 | 108.80 |  |  |  |
|  |  | 1:1000 | 93.02 | 94.60 | 98.33 |  |  |  |
| **65** | **Polychaete, *Travisia sp*.** | 1:100 | 216.13 | 126.24 | **171.20** | **0.0096** |  |  |
|  |  | 1:100 | 205.41 | 114.85 | 178.85 |  |  |  |
|  |  | 1:300 | 107.63 | 112.77 | 95.44 |  |  |  |
|  |  | 1:300 | 99.71 | 117.08 | 85.16 |  |  |  |
|  |  | 1:1000 | 100.64 | 123.84 | 81.26 |  |  |  |
|  |  | 1:1000 | 78.64 | 101.50 | 77.47 |  |  |  |
| **93** | **Large polychaete** | 1:100 | 23.18 | 92.42 | **25.08** | **0.0002** |  |  |
|  |  | 1:300 | 50.11 | 109.93 | **45.59** |  |  |  |
|  |  | 1:1000 | 86.75 | 104.09 | 83.35 |  |  |  |
|  |  | 1:100 | 16.17 | 167.97 | **9.63** |  |  |  |
|  |  | 1:200 | 37.64 | 130.82 | **28.77** |  |  |  |
|  |  | 1:400 | 69.01 | 117.72 | 58.62 |  |  |  |
| 40 | Large sipunculid worm | 1:100 | 63.91 | 80.65 | 79.24 |  |  |  |
|  |  | 1:300 | 50.45 | 99.13 | 50.89 |  |  |  |
|  |  | 1:1000 | 63.16 | 99.16 | 63.70 |  |  |  |
| 81 | Large sipunculid worm | 1:100 | 104.97 | 91.77 | 114.38 |  |  |  |
|  |  | 1:100 | 97.66 | 83.72 | 116.66 |  |  |  |
|  |  | 1:300 | 96.85 | 93.63 | 103.43 |  |  |  |
|  |  | 1:300 | 93.86 | 103.60 | 90.60 |  |  |  |
|  |  | 1:1000 | 96.24 | 94.91 | 101.41 |  |  |  |
|  |  | 1:1000 | 100.73 | 114.40 | 88.04 |  |  |  |
| 11 | Gorgonian coral | 1:100 | 91.03 | 100.38 | 90.69 |  |  |  |
|  |  | 1:100 | 104.06 | 92.68 | 112.28 |  |  |  |
|  |  | 1:300 | 92.79 | 92.66 | 100.13 |  |  |  |
|  |  | 1:300 | 99.37 | 97.89 | 101.51 |  |  |  |
|  |  | 1:1000 | 101.72 | 106.06 | 95.91 |  |  |  |
|  |  | 1:1000 | 115.28 | 144.38 | 79.85 |  |  |  |
| 25 | Coral (o. *Corallimorpharia*) | 1:100 | 94.89 | 85.07 | 111.54 |  |  |  |
|  |  | 1:100 | 88.13 | 94.75 | 93.01 |  |  |  |
|  |  | 1:300 | 92.86 | 104.77 | 88.63 |  |  |  |
|  |  | 1:300 | 97.14 | 93.80 | 103.55 |  |  |  |
| 91 | Coral, *Eunephthya sp*. | 1:100 | 56.10 | 94.99 | 59.07 |  |  |  |
|  |  | 1:300 | 63.00 | 120.34 | 52.35 |  |  |  |
|  |  | 1:1000 | 88.99 | 110.87 | 80.27 |  |  |  |
| 27 | Sea urchin, *Cystechinus sp*., gonads | 1:100 | 88.61 | 101.24 | 87.53 |  |  |  |
|  |  | 1:100 | 107.83 | 89.39 | 120.63 |  |  |  |
|  |  | 1:300 | 92.38 | 94.14 | 98.12 |  |  |  |
|  |  | 1:300 | 108.54 | 93.92 | 115.57 |  |  |  |
|  |  | 1:1000 | 104.65 | 97.45 | 107.39 |  |  |  |
|  |  | 1:1000 | 112.15 | 104.83 | 106.98 |  |  |  |
| 45 | Sea urchin, *Cystechinus sp*., gonads | 1:100 | 107.44 | 90.28 | 119.01 |  |  |  |
|  |  | 1:100 | 31.89 | 50.27 | 63.43 |  |  |  |
|  |  | 1:300 | 104.93 | 95.85 | 109.48 |  |  |  |
|  |  | 1:300 | 106.71 | 95.02 | 112.30 |  |  |  |
|  |  | 1:1000 | 107.26 | 90.25 | 118.84 |  |  |  |
|  |  | 1:1000 | 102.47 | 95.61 | 107.17 |  |  |  |
| 56 | Sea urchin, *Cystechinus sp*., gonads | 1:100 | 27.38 | 42.14 | 64.98 |  |  |  |
|  |  | 1:100 | 101.73 | 90.42 | 112.52 |  |  |  |
|  |  | 1:300 | 128.71 | 102.47 | 125.61 |  |  |  |
|  |  | 1:300 | 91.87 | 96.25 | 95.45 |  |  |  |
|  |  | 1:1000 | 108.41 | 104.57 | 103.67 |  |  |  |
|  |  | 1:1000 | 104.51 | 100.07 | 104.44 |  |  |  |
| 30 | Demosponge №8 | 1:100 | 105.46 | 86.53 | 121.87 |  |  |  |
|  |  | 1:100 | 97.88 | 90.35 | 108.33 |  |  |  |
|  |  | 1:300 | 90.69 | 96.71 | 93.77 |  |  |  |
|  |  | 1:300 | 98.02 | 97.08 | 100.98 |  |  |  |
|  |  | 1:1000 | 109.79 | 94.14 | 116.62 |  |  |  |
|  |  | 1:1000 | 107.09 | 102.29 | 104.69 |  |  |  |
| 68 | Demosponge №8 | 1:100 | 91.01 | 85.66 | 106.24 |  |  |  |
|  |  | 1:100 | 100.04 | 101.69 | 98.37 |  |  |  |
|  |  | 1:300 | 103.36 | 92.86 | 111.31 |  |  |  |
|  |  | 1:300 | 98.24 | 97.52 | 100.74 |  |  |  |
|  |  | 1:1000 | 100.20 | 103.13 | 97.16 |  |  |  |
|  |  | 1:1000 | 108.75 | 100.16 | 108.58 |  |  |  |
| 33 | Demosponge № 7 | 1:100 | 63.35 | 88.17 | 71.85 |  |  |  |
|  |  | 1:100 | 50.30 | 78.11 | 64.40 |  |  |  |
|  |  | 1:300 | 103.10 | 93.39 | 110.40 |  |  |  |
|  |  | 1:300 | 107.81 | 103.96 | 103.71 |  |  |  |
|  |  | 1:1000 | 114.43 | 93.77 | 122.02 |  |  |  |
|  |  | 1:1000 | 103.90 | 91.93 | 113.02 |  |  |  |
| 99 | Demosponge № 7 | 1:100 | 105.51 | 88.66 | 119.00 |  |  |  |
|  |  | 1:100 | 97.60 | 86.32 | 113.06 |  |  |  |
|  |  | 1:300 | 98.66 | 92.54 | 106.61 |  |  |  |
|  |  | 1:300 | 99.02 | 100.13 | 98.89 |  |  |  |
| 43 | Demosponge №5 | 1:100 | 90.84 | 86.86 | 104.58 |  |  |  |
|  |  | 1:100 | 98.71 | 97.16 | 101.60 |  |  |  |
|  |  | 1:300 | 97.06 | 93.29 | 104.04 |  |  |  |
|  |  | 1:300 | 82.64 | 104.71 | 78.92 |  |  |  |
|  |  | 1:1000 | 104.34 | 79.16 | 131.80 |  |  |  |
|  |  | 1:1000 | 90.80 | 96.33 | 94.25 |  |  |  |
| 57 | Demosponge № 6 | 1:100 | 66.01 | 105.52 | 62.56 |  |  |  |
|  |  | 1:100 | 86.53 | 93.37 | 92.68 |  |  |  |
|  |  | 1:300 | 111.40 | 87.41 | 127.44 |  |  |  |
|  |  | 1:300 | 97.44 | 97.85 | 99.58 |  |  |  |
|  |  | 1:1000 | 111.38 | 111.06 | 100.28 |  |  |  |
|  |  | 1:1000 | 86.36 | 84.70 | 101.96 |  |  |  |
| **83** | **Demosponge № 6** | 1:100 | 39.50 | 103.84 | **38.04** | **0.0002** |  |  |
|  |  | 1:300 | 54.84 | 121.02 | **45.32** |  |  |  |
|  |  | 1:1000 | 93.10 | 93.59 | 99.48 |  |  |  |
|  |  | 1:100 | 42.00 | 139.53 | **30.10** |  |  |  |
|  |  | 1:200 | 70.75 | 128.08 | 55.24 |  |  |  |
|  |  | 1:400 | 98.07 | 128.91 | 76.07 |  |  |  |
| 89 | Demosponge № 6 | 1:100 | 154.15 | 109.54 | 140.72 |  |  |  |
|  |  | 1:100 | 143.61 | 105.51 | 136.11 |  |  |  |
|  |  | 1:300 | 106.03 | 114.44 | 92.65 |  |  |  |
|  |  | 1:300 | 100.29 | 97.59 | 102.77 |  |  |  |
|  |  | 1:1000 | 111.86 | 99.26 | 112.70 |  |  |  |
|  |  | 1:1000 | 103.41 | 95.21 | 108.61 |  |  |  |
| 34 | Hexactinellid sponge | 1:100 | 91.01 | 116.37 | 78.21 |  |  |  |
|  |  | 1:100 | 99.46 | 113.96 | 87.28 |  |  |  |
|  |  | 1:300 | 102.43 | 99.19 | 103.27 |  |  |  |
|  |  | 1:300 | 95.22 | 116.66 | 81.63 |  |  |  |
|  |  | 1:1000 | 106.17 | 101.99 | 104.10 |  |  |  |
|  |  | 1:1000 | 111.90 | 120.30 | 93.02 |  |  |  |
| **39** | **Hexactinellid sponge** | 1:100 | 30.78 | 70.67 | **43.55** | **0.0008** |  |  |
|  |  | 1:300 | 45.13 | 85.94 | 52.51 |  |  |  |
|  |  | 1:1000 | 62.82 | 79.97 | 78.55 |  |  |  |
|  |  | 1:100 | 60.55 | 111.36 | 54.37 |  |  |  |
|  |  | 1:200 | 78.04 | 121.21 | 64.38 |  |  |  |
|  |  | 1:400 | 104.37 | 115.57 | 90.31 |  |  |  |
| 71 | Hexactinellid sponge | 1:100 | 104.90 | 107.58 | 97.51 |  |  |  |
|  |  | 1:100 | 102.19 | 100.53 | 101.65 |  |  |  |
|  |  | 1:300 | 97.77 | 113.54 | 86.11 |  |  |  |
|  |  | 1:300 | 88.69 | 93.04 | 95.32 |  |  |  |
|  |  | 1:1000 | 103.22 | 97.58 | 105.77 |  |  |  |
|  |  | 1:1000 | 107.43 | 80.78 | 132.99 |  |  |  |
| 74 | Hexactinellid sponge | 1:100 | 115.43 | 114.55 | 100.77 |  |  |  |
|  |  | 1:200 | 104.45 | 94.30 | 110.76 |  |  |  |
|  |  | 1:400 | 105.29 | 96.95 | 108.60 |  |  |  |
| 82 | Hexactinellid sponge, *Hyalonema sp*. | 1:100 | 56.39 | 72.32 | 77.98 |  |  |  |
|  |  | 1:300 | 76.83 | 100.94 | 76.12 |  |  |  |
|  |  | 1:1000 | 88.22 | 93.47 | 94.38 |  |  |  |
| 8 | Sea pen, *Umbellula sp*. | 1:100 | 116.49 | 100.84 | 115.52 |  |  |  |
|  |  | 1:100 | 111.18 | 88.46 | 125.69 |  |  |  |
|  |  | 1:300 | 95.37 | 95.48 | 99.88 |  |  |  |
|  |  | 1:300 | 110.27 | 101.53 | 108.61 |  |  |  |
|  |  | 1:1000 | 100.64 | 107.16 | 93.92 |  |  |  |
|  |  | 1:1000 | 116.57 | 96.73 | 120.51 |  |  |  |
| 35 | Sea pen, *Umbellula sp*. | 1:100 | 113.56 | 87.18 | 130.26 |  |  |  |
|  |  | 1:100 | 97.59 | 97.63 | 99.96 |  |  |  |
|  |  | 1:300 | 108.37 | 96.07 | 112.81 |  |  |  |
|  |  | 1:300 | 96.10 | 101.53 | 94.65 |  |  |  |
|  |  | 1:1000 | 107.71 | 102.03 | 105.56 |  |  |  |
|  |  | 1:1000 | 124.24 | 104.82 | 118.52 |  |  |  |
| 87 | Sea pen, *Umbellula sp*. | 1:100 | 89.96 | 103.51 | 86.91 |  |  |  |
|  |  | 1:100 | 106.38 | 90.51 | 117.54 |  |  |  |
|  |  | 1:300 | 92.02 | 111.41 | 82.60 |  |  |  |
|  |  | 1:300 | 86.64 | 101.01 | 85.77 |  |  |  |
|  |  | 1:1000 | 108.03 | 52.45 | 205.96 |  |  |  |
|  |  | 1:1000 | 97.41 | 97.42 | 99.99 |  |  |  |
| 1 | White echiuran | 1:100 | 98.82 | 86.69 | 113.99 |  |  |  |
|  |  | 1:100 | 109.80 | 94.56 | 116.12 |  |  |  |
|  |  | 1:300 | 93.31 | 106.89 | 87.29 |  |  |  |
|  |  | 1:300 | 109.15 | 92.25 | 118.32 |  |  |  |
|  |  | 1:1000 | 93.60 | 94.29 | 99.27 |  |  |  |
|  |  | 1:1000 | 111.52 | 145.35 | 76.72 |  |  |  |
| 73 | White echiuran | 1:100 | 104.38 | 101.25 | 103.09 |  |  |  |
|  |  | 1:100 | 103.41 | 92.95 | 111.26 |  |  |  |
|  |  | 1:300 | 98.23 | 93.51 | 105.05 |  |  |  |
|  |  | 1:300 | 101.08 | 97.34 | 103.84 |  |  |  |
| 48 | Deep-sea shrimp, *Hymenodora glacialis* | 1:100 | 140.46 | 161.89 | 86.76 |  |  |  |
|  |  | 1:200 | 110.89 | 126.08 | 87.96 |  |  |  |
|  |  | 1:400 | 115.96 | 114.60 | 101.19 |  |  |  |
| **52** | **Deep-sea shrimp, *Hymenodora glacialis*** | 1:100 | 18.70 | 66.35 | **28.19** | **0.001** |  |  |
|  |  | 1:300 | 57.55 | 82.96 | 69.37 |  |  |  |
|  |  | 1:1000 | 71.40 | 75.41 | 94.69 |  |  |  |
|  |  | 1:100 | 17.42 | 25.82 | 67.46 |  |  |  |
|  |  | 1:200 | 65.63 | 128.29 | 51.15 |  |  |  |
|  |  | 1:400 | 102.40 | 111.88 | 91.53 |  |  |  |
| 62 | Deep-sea shrimp, *Hymenodora glacialis* | 1:100 | 77.50 | 92.83 | 83.49 |  |  |  |
|  |  | 1:300 | 97.59 | 146.94 | 66.42 |  |  |  |
|  |  | 1:1000 | 95.80 | 107.05 | 89.49 |  |  |  |
|  |  | 1:100 | 88.14 | 107.13 | 82.28 |  |  |  |
|  |  | 1:200 | 113.37 | 97.23 | 116.60 |  |  |  |
|  |  | 1:400 | 125.03 | 88.89 | 140.65 |  |  |  |
| **80** | **Deep-sea shrimp, *Hymenodora glacialis*** | 1:100 | 144.45 | 92.43 | **156.27** | **0.0007** |  |  |
|  |  | 1:100 | 131.74 | 87.44 | **150.67** |  |  |  |
|  |  | 1:300 | 98.71 | 99.84 | 98.86 |  |  |  |
|  |  | 1:300 | 98.06 | 99.00 | 99.06 |  |  |  |
|  |  | 1:1000 | 108.98 | 102.69 | 106.13 |  |  |  |
|  |  | 1:1000 | 111.59 | 96.30 | 115.88 |  |  |  |
| 67 | Skeleton shrimp, *Caprella sp*. | 1:100 | 92.77 | 88.69 | 104.60 |  |  |  |
|  |  | 1:100 | 87.08 | 104.16 | 83.60 |  |  |  |
|  |  | 1:300 | 91.93 | 94.13 | 97.67 |  |  |  |
|  |  | 1:300 | 99.84 | 95.25 | 104.82 |  |  |  |
|  |  | 1:1000 | 103.49 | 93.58 | 110.59 |  |  |  |
|  |  | 1:1000 | 113.78 | 100.03 | 113.75 |  |  |  |
| 85 | Isopod crustacean | 1:100 | 87.49 | 158.46 | 55.21 |  |  |  |
|  |  | 1:200 | 114.45 | 136.62 | 83.78 |  |  |  |
|  |  | 1:400 | 136.73 | 101.23 | 135.07 |  |  |  |
| **96** | **Crab, *Munidopsis antonii*, hepatopancreas** | 1:100 | 10.36 | 102.81 | **10.08** | **0.001** |  |  |
|  |  | 1:300 | 21.88 | 124.93 | **17.52** |  |  |  |
|  |  | 1:1000 | 61.69 | 98.30 | 62.75 |  |  |  |
|  |  | 1:100 | 4.90 | 159.98 | **3.06** |  |  |  |
|  |  | 1:200 | 21.48 | 151.78 | **14.15** |  |  |  |
|  |  | 1:400 | 55.35 | 131.51 | **42.09** |  |  |  |
| **97** | **Decapod crustacean, *Calocarides quinqueseriatus*, hepatopancreas** | 1:100 | 3.52 | 86.05 | **4.09** | **0.0031** |  |  |
|  |  | 1:300 | 9.75 | 103.02 | **9.47** |  |  |  |
|  |  | 1:1000 | 39.95 | 81.93 | **48.76** |  |  |  |
|  |  | 1:100 | 2.60 | 109.74 | **2.37** |  |  |  |
|  |  | 1:200 | 7.75 | 151.55 | **5.11** |  |  |  |
|  |  | 1:400 | 16.51 | 132.67 | **12.44** |  |  |  |
| 46 | Decapod crustacean, *Calocarides quinqueseriatus*, muscles | 1:100 | 80.04 | 119.91 | 66.74 | 0.0878 |  |  |
|  |  | 1:200 | 69.78 | 121.67 | 57.35 |  |  |  |
|  |  | 1:400 | 54.05 | 96.19 | 56.19 |  |  |  |
| 13 | Colonial ascidian | 1:100 | 63.84 | 68.63 | 93.02 |  |  |  |
|  |  | 1:300 | 74.43 | 107.97 | 68.94 |  |  |  |
|  |  | 1:1000 | 99.76 | 105.76 | 94.33 |  |  |  |
| 79 | Colonial ascidian | 1:100 | 101.45 | 87.68 | 115.70 |  |  |  |
|  |  | 1:100 | 108.33 | 101.37 | 106.87 |  |  |  |
|  |  | 1:300 | 104.15 | 97.05 | 107.31 |  |  |  |
|  |  | 1:300 | 97.27 | 99.83 | 97.43 |  |  |  |
|  |  | 1:1000 | 100.57 | 98.13 | 102.49 |  |  |  |
|  |  | 1:1000 | 75.54 | 99.01 | 76.30 |  |  |  |
| 92 | Colonial ascidian | 1:100 | 96.28 | 92.26 | 104.35 |  |  |  |
|  |  | 1:100 | 104.14 | 88.16 | 118.13 |  |  |  |
|  |  | 1:300 | 87.79 | 96.60 | 90.88 |  |  |  |
|  |  | 1:300 | 102.59 | 96.33 | 106.51 |  |  |  |
|  |  | 1:1000 | 78.85 | 101.41 | 77.75 |  |  |  |
|  |  | 1:1000 | 110.49 | 99.32 | 111.24 |  |  |  |
| 16 | Actinia, *Phelliactis callicyclus* | 1:100 | 286.46 | 104.40 | **274.40** | 0.1162 | **0.048** | ***Phelliactis callicyclus* altogether** |
|  |  | 1:300 | 86.79 | 115.64 | 75.05 |  |  |  |
|  |  | 1:1000 | 95.25 | 91.15 | 104.50 |  | **0.0197** | **Actinias altogether** |
|  |  | 1:100 | 176.99 | 157.99 | 112.02 |  |  |  |
|  |  | 1:200 | 107.63 | 133.24 | 80.78 |  |  |  |
|  |  | 1:400 | 92.87 | 103.16 | 90.02 |  |  |  |
| 44 | Actinia, *Phelliactis callicyclus* | 1:100 | 84.76 | 73.70 | 115.01 | **0.0005** |  |  |
|  |  | 1:100 | 80.21 | 63.87 | 125.58 |  |  |  |
|  |  | 1:300 | 94.27 | 88.53 | 106.49 |  |  |  |
|  |  | 1:300 | 102.00 | 100.33 | 101.66 |  |  |  |
|  |  | 1:1000 | 95.08 | 98.29 | 96.74 |  |  |  |
|  |  | 1:1000 | 108.32 | 107.99 | 100.31 |  |  |  |
| 55 | Actinia | 1:100 | 321.33 | 76.87 | **418.03** | 0.1505 |  |  |
|  |  | 1:300 | 70.73 | 110.83 | 63.82 |  |  |  |
|  |  | 1:1000 | 77.79 | 87.97 | 88.43 |  |  |  |
|  |  | 1:100 | 3.40 | 11.46 | 29.63 |  |  |  |
|  |  | 1:200 | 511.24 | 159.12 | **321.28** |  |  |  |
|  |  | 1:400 | 131.00 | 134.41 | 97.47 |  |  |  |
| 78 | Actinia | 1:100 | 212.00 | 115.02 | **184.32** | 0.2261 |  |  |
|  |  | 1:200 | 92.48 | 121.68 | 76.00 |  |  |  |
|  |  | 1:400 | 90.33 | 110.89 | 81.46 |  |  |  |
| **22** | **Brittle star, *Ophiura irrorata*** | 1:100 | 5.30 | 111.25 | **4.76** | **0.0015** | **<0.0001** | **Ophiuras altogether** |
|  |  | 1:300 | 29.13 | 128.93 | **22.59** |  |  |  |
|  |  | 1:1000 | 81.34 | 84.04 | 96.78 |  |  |  |
|  |  | 1:100 | 0.71 | 117.72 | **0.61** |  |  |  |
|  |  | 1:200 | 2.78 | 112.55 | **2.47** |  |  |  |
|  |  | 1:400 | 19.96 | 122.91 | **16.24** |  |  |  |
| **47** | **Brittle star, *Ophiura irrorata*** | 1:100 | 6.32 | 88.14 | **7.17** | **0.0001** |  |  |
|  |  | 1:300 | 34.06 | 90.38 | **37.69** |  |  |  |
|  |  | 1:1000 | 63.14 | 84.40 | 74.81 |  |  |  |
|  |  | 1:100 | 1.87 | 118.30 | **1.58** |  |  |  |
|  |  | 1:200 | 23.09 | 109.51 | **21.09** |  |  |  |
|  |  | 1:400 | 60.65 | 112.80 | 53.77 |  |  |  |
| **38** | **Brittle star, *Ophiura sp*. №1** | 1:100 | 3.23 | 114.66 | **2.82** | **0.0006** |  |  |
|  |  | 1:300 | 45.14 | 125.32 | **36.02** |  |  |  |
|  |  | 1:1000 | 104.25 | 91.58 | 113.84 |  |  |  |
|  |  | 1:100 | 1.06 | 91.51 | **1.16** |  |  |  |
|  |  | 1:200 | 6.44 | 120.61 | **5.34** |  |  |  |
|  |  | 1:400 | 55.32 | 151.95 | **36.41** |  |  |  |
| **98** | **Brittle star, *Ophiura sp*. №1** | 1:100 | 0.32 | 10.35 | **3.11** | **0.0494** |  |  |
|  |  | 1:200 | 1.37 | 116.56 | **1.17** |  |  |  |
|  |  | 1:400 | 2.50 | 118.67 | **2.11** |  |  |  |
| **76** | **Brittle star, *Ophiura sp*. №2** | 1:100 | 1.66 | 76.73 | **2.16** | **0.002** |  |  |
|  |  | 1:300 | 13.41 | 91.46 | **14.67** |  |  |  |
|  |  | 1:1000 | 44.73 | 81.06 | 55.18 |  |  |  |
|  |  | 1:100 | 0.32 | 7.46 | **4.30** |  |  |  |
|  |  | 1:200 | 1.65 | 76.50 | **2.16** |  |  |  |
|  |  | 1:400 | 23.41 | 106.17 | **22.04** |  |  |  |
| 18 | Sea pig, *Scotoplanes aff. theeli*, skin and muscular sac | 1:100 | 103.64 | 91.15 | 113.70 |  |  |  |
|  |  | 1:100 | 100.63 | 93.22 | 107.96 |  |  |  |
|  |  | 1:300 | 108.78 | 105.87 | 102.74 |  |  |  |
|  |  | 1:300 | 94.65 | 101.07 | 93.65 |  |  |  |
|  |  | 1:1000 | 100.06 | 91.26 | 109.64 |  |  |  |
|  |  | 1:1000 | 102.11 | 98.09 | 104.10 |  |  |  |
| 19 | Sea pig, *Scotoplanes aff. theeli*, gonads | 1:100 | 122.77 | 119.96 | 102.35 |  |  |  |
|  |  | 1:200 | 103.63 | 110.84 | 93.49 |  |  |  |
|  |  | 1:400 | 95.84 | 108.90 | 88.00 |  |  |  |
| 29 | Sea pig, *Scotoplanes aff. theeli*, gonads | 1:100 | 129.26 | 89.72 | 144.08 |  |  |  |
|  |  | 1:300 | 89.23 | 101.52 | 87.89 |  |  |  |
|  |  | 1:1000 | 102.07 | 80.01 | 127.57 |  |  |  |
|  |  | 1:100 | 100.79 | 123.08 | 81.89 |  |  |  |
|  |  | 1:200 | 118.48 | 123.82 | 95.69 |  |  |  |
|  |  | 1:400 | 101.89 | 114.34 | 89.11 |  |  |  |
| 32 | Sea pig, *Scotoplanes aff. theeli*, gonads | 1:100 | 48.66 | 115.27 | 42.22 | 0.121 |  |  |
|  |  | 1:300 | 62.53 | 114.78 | 54.48 |  |  |  |
|  |  | 1:1000 | 80.92 | 110.32 | 73.35 |  |  |  |
|  |  | 1:100 | 91.54 | 115.82 | 79.03 |  |  |  |
|  |  | 1:200 | 102.52 | 88.66 | 115.63 |  |  |  |
|  |  | 1:400 | 149.63 | 115.07 | 130.04 |  |  |  |
| 36 | Sea pig, *Scotoplanes aff. theeli* | 1:100 | 103.62 | 94.16 | 110.04 |  |  |  |
|  |  | 1:100 | 100.51 | 110.11 | 91.28 |  |  |  |
|  |  | 1:300 | 103.88 | 98.39 | 105.58 |  |  |  |
|  |  | 1:300 | 77.15 | 94.31 | 81.80 |  |  |  |
|  |  | 1:1000 | 113.30 | 100.05 | 113.24 |  |  |  |
|  |  | 1:1000 | 103.24 | 101.33 | 101.89 |  |  |  |
